# Cardiac fibroblast activation detected by Ga-68 FAPI PET imaging as a potential novel biomarker of cardiac injury/remodeling

**DOI:** 10.1007/s12350-020-02307-w

**Published:** 2020-09-25

**Authors:** J. Siebermair, M. I. Köhler, J. Kupusovic, S. G. Nekolla, L. Kessler, J. Ferdinandus, N. Guberina, M. Stuschke, H. Grafe, J. T. Siveke, S. Kochhäuser, W. P. Fendler, M. Totzeck, R. Wakili, L. Umutlu, T. Schlosser, T. Rassaf, C. Rischpler

**Affiliations:** 1 Department of Cardiology and Vascular Medicine, University of Essen Medical School, West German Heart and Vascular Center Essen, University Duisburg-Essen, Hufelandstr. 55, 45147 Essen, Germany; 2 Department of Nuclear Medicine, School of Medicine, Technische Universität München, Munich, Germany; 3 DZHK (Deutsches Zentrum für Herz-Kreislauf-Forschung e.V.), Partner Site Munich Heart Alliance, Munich, Germany; 4 Department of Nuclear Medicine, Medical Faculty, University Hospital Essen, University of Duisburg-Essen, Hufelandstr. 55, 45147 Essen, Germany; 5 Department of Radiotherapy, University Hospital Essen, University Duisburg-Essen, Essen, Germany; 6 Institute for Developmental Cancer Therapeutics, West German Cancer Center, University Hospital Essen, Hufelandstrasse 55, 45147 Essen, Germany; 7 Division of Solid Tumor Translational Oncology, German Cancer Consortium (DKTK, partner site Essen) and German Cancer Research Center, DKFZ, Im Neuenheimer Feld 280, 69120 Heidelberg, Germany; 8 University Hospital Essen, Institute for Diagnostic and Interventional Radiology and Neuroradiology, University of Duisburg-Essen, Hufelandstr. 55, 45147 Essen, Germany

**Keywords:** PET, CT, MRI, myocardial ischemia and infarction, heart failure

## Abstract

**Background:**

Fibroblast activation protein (FAP) as a specific marker of activated fibroblasts can be visualized by positron emission tomography (PET) using Ga-68-FAP inhibitors (FAPI). Gallium-68-labeled FAPI is increasingly used in the staging of various cancers. In addition, the first cases of theranostic approaches have been reported. In this work, we describe the phenomenon of myocardial FAPI uptake in patients who received a Ga-68 FAPI PET for tumor staging.

**Method and results:**

Ga-68 FAPI PET examinations for cancer staging were retrospectively analyzed with respect to cardiac tracer uptake. Standardized uptake values (SUV) were correlated to clinical covariates in a univariate regression model.

From 09/2018 to 11/2019 N = 32 patients underwent FAPI PET at our institution. Six out of 32 patients (18.8%) demonstrated increased localized myocardial tracer accumulation, with remote FAPI uptake being significantly higher in patients with vs without localized focal myocardial uptake (SUV_max_ 2.2 ± .6 vs 1.5 ± .4, *P* < .05 and SUV_mean_ 1.6 ± .4 vs 1.2 ± .3, *P* < .05, respectively). Univariate regression demonstrated a significant correlation of coronary artery disease (CAD), age and left ventricular ejection fraction (LVEF) with remote SUV_mean_ uptake, the latter with a very strong correlation with remote uptake (*R*^2^ = .74, *P* < .01).

**Conclusion:**

Our study indicates an association of CAD, age, and LVEF with FAPI uptake. Further studies are warranted to assess if fibroblast activation can be reliably measured and may be used for risk stratification regarding early detection or progression of CAD and left ventricular remodeling.

**Electronic supplementary material:**

The online version of this article (10.1007/s12350-020-02307-w) contains supplementary material, which is available to authorized users.

## Spanish Abstract

**Antecedentes:**

Proteína de activación de fibroblastos (FAP) como marcador específico de fibroblastos maduros activados se puede visualizar mediante tomografía por emisión de positrones (PET) usando inhibidores de Ga-68-FAP (FAPI).

El FAPI marcado con galio 68 se usa cada vez más en la estatificación de varios tipos de cáncer.Además, se han reportado los primeros casos de abordajes teranósticos.

En este trabajo describimos el fenómeno de la captación de FAPI miocárdica en pacientes que recibieron Ga-68 FAPI PET para estatificación tumoral.

**Método y resultados:**

Los exámenes de PET Ga-68 FAPI para estadificación de cáncer se analizaron retrospectivamente con respecto a la captación del marcador cardíaco. Los valores de absorción estandarizados (SUV) se correlacionaron con covariables clínicas en un modelo de regresión univariante.

Del 09/2018 al 11/2019 con una n = 32 pacientes fueron sometidos a PET FAPI en nuestra institución. Seis de 32 pacientes (18.8%) demostraron un aumento de acumulación del marcador localizado en el miocardio, con la captación remota de FAPI siendo significativamente mayor en pacientes con aumento de la captación vs sin captación focalizada de miocardio (SUVmax 2.2 ± 0.6 vs. 1.5 ± 0.4, p <0.05 y SUV mean 1.6 ± 0.4 vs. 1.2 ± 0.3, p <0.05, respectivamente).

La regresión univariante demostró una correlación significativa de la enfermedad de la arteria coronaria (CAD), la edad y la fracción de eyección ventricular izquierda (FEVI) con absorción SUV remota, esta última con una muy fuerte correlación con la captación remota (R² = 0.74, p <0.01).

**Conclusión:**

Nuestro estudio indica una asociación de CAD, edad y FEVI con la captación de FAPI.

Se necesitan más estudios para evaluar si la activación de fibroblastos se puede medir de manera confiable y se puede usar para la estratificación de riesgo con respecto a la detección temprana o la progresión de la CAD y la remodelación ventricular izquierda.

## Chinese Abstract

****背景**:**

成纤维细胞活化蛋白(FAP)作为活化成纤维细胞的一种特异性标志物,可以通过Ga-68-FAP抑制剂(FAPI)的正电子发射断层扫描(PET)显像进行活体可视化。Ga-68标记的FAPI已被越来越多地用于各种癌症的分期。并且已有第一个诊疗一体化的病例报道。本文在接受Ga-68 FAPI PET进行肿瘤分期患者中研究了心肌FAPI摄取结果。

****方法和结果**:**

回顾性分析了Ga-68 FAPI PET检查癌症分期的心脏示踪剂摄取情况。摄取值(SUV)作为因变量,临床资料作为协变量,纳入单变量回归模型。

从2018年9月到2019年11月,共纳入32例在我院接受了FAPI PET检查的患者。其中6例患者(18.8%)表现为局灶性心肌摄取。与无局灶性心肌摄取的患者相比,有局灶性摄取患者远处心肌FAPI摄取值更高(SUV_max_ 2.2±0.6 vs. 1.5±0.4,P<0.05,SUV_mean_ 1.6±0.4 vs. 1.2±0.3,P<0.05)。单因素回归分析表明,冠状动脉疾病(CAD)病史、年龄和左心室射血分数(LVEF)与远处心肌SUV_mean_摄取显著相关,其中LVEF的相关性最高(R²=0.74,P<0.01)。

****结论**:**

本研究表明,CAD病史、年龄和LVEF与FAPI的摄取值有关。需要进一步的研究评估FAPI能否可靠地测定成纤维细胞的活化,并用于CAD的早期诊断或进展以及左心室重构的风险分层。

## French Abstract

**Contexte:**

La protéine d’activation des fibroblastes (FAP) activés et matures peut être visualisée par tomographie à émission de positons (TEP) à l’aide d’inhibiteurs de l’activation des fibroblastes (FAPI). FAPI marqué au gallium 68 est de plus en plus utilisé dans la stratification de divers cancers. De plus, les premiers exemples d’approches théranostiques ont été rapportés. Dans ce travail nous décrivons la captation myocardique de FAPI chez les patients qui bénéficié d’une TEP au Ga-68 FAPI pour stratification tumorale.

**Méthode et résultats:**

Les examens TEP Ga-68 FAPI pour la stratification oncologique ont été analysés rétrospectivement pour l’absorption du traceur au niveau cardiaque. Les valeurs d’absorption normalisées (SUV) font été corrélées aux variables cliniques selon un modèle de régression univarié. A partir de septembre 2018 jusqu’en novembre 2019, 32 patients ont bénéficié d’une TEP FAPI dans notre établissement. Six de nos 32 patients (18,8%) ont démontré une augmentation focale de captation du tracer au niveau myocardique. Les foyers systémiques se sont révélés significativement plus élevé chez les patients avec foyers myocardiques localisés (SUV max 2,2 ± 0,6 vs 1,5 ± 0,4, p <0,05 et SUV mean 1,6 ± 0,4 vs 1,2 ± 0,3, p <0,05, respectivement). Nous avons observé une corrélation significative entre la maladie coronarienne, l’âge, la fraction d’éjection du ventricule gauche et la présence de foyer myocardiques FAPI (R² = 0,74, p <0,01)

**Conclusion:**

Notre étude indique une association entre la maladie cardiovasculaire coronarienne, l’âge et la FEVG et la captation myocardique de FAPI. Des études additionnelles sont nécessaires pour déterminer si l’activation des fibroblastes peut être mesurée de manière fiable et utilisée pour la détection et la progression de la maladie coronarienne et le remodelage du ventricule gauche

## Introduction

Activation of fibroblasts is mandatory for repair and regeneration after myocardial injury including myocardial infarction (MI), progressive heart failure, or chemotherapy-induced injury, with the main mechanism of extracellular matrix remodeling.^1^,^2^ Several pathways and mediators are suggested to play a role in fibrosis development, among others the renin-angiotensin-aldosterone (RAA) system, fibrogenic growth factors like transforming growth factor beta (TGF-β), or inflammatory cytokines and chemokines.^3^,^4^ In addition, fibroblast activation protein (FAP) alpha has been demonstrated to be a specific marker of activated mature fibroblasts.^5^ The fibroblast activation protein, also known as dipeptidyl peptidase (DPP) 4, is an enzyme and represents a serine proteinase. During embryonic development, FAP expression can be detected in a variety of tissues.^6^ However, in adult humans, intensive expression is only found in the setting of wound healing, in fibrotic remodeling processes like in liver fibrosis and cirrhosis^7^ and in the stroma of the majority of malignant cancer types.^8^–^10^ The highest FAPI uptake was reported in sarcoma, esophageal, breast and lung cancer, and cholangiocarcinoma pointing at the dominant expression of FAP in solid tumors.^9^ A study analyzing brain samples could even demonstrate increased FAP expression in high-grade gliomas.^11^ For this reason, it is not only a very promising target in the diagnosis and potentially also in radio-ligand therapy of various tumor types, but it also represents a highly interesting target to diagnose and monitor tissue alterations such as remodeling processes due to different noxious agents including ischemia, chemo-, or radiation therapy. Recently, a Ga-68-labeled tracer has been developed, which has already been increasingly used in the diagnosis and staging of malignancy.^12^ The quinolone-based FAP inhibitors can be labeled using the chelator DOTA. Of the Ga-68-labeled FAP inhibitors described, FAPI-04 has been considered the most promising one due to its characteristics such as low nano-molar affinity, an almost complete internalization of more than 90% and rapid blood clearance. Advantages of FAPI over FDG are, among other things, that it represents a more specific signal with very low uptake in healthy organs and thus a very low background, which also provided the possibility for first theranostic concepts using Y-90 FAPI.^12^ Furthermore, FAPI could be used in tumors that often have no or only limited increased FDG uptake resulting in a low sensitivity, such as pancreatic cancer or hepatocellular carcinoma.^13^ As there is no approved tracer so far, FAPI can only be used on a compassionate use basis. As many aspects of ventricular fibrosis development are still unclear,^3^ this novel imaging technique has the potential to significantly improve the understanding of ventricular fibrosis development under various clinical settings (e.g., cardiac side effects of modern cancer therapy). The latter consists of radio-, conventional chemo-, immuno-, and targeted therapy.^14^ Heart failure is among the most common side effects of these multimodal approaches and has been related to the development of fibrosis.^15^ Early detection of myocardial remodeling and fibrosis may be critical to prevent development of overt heart failure.

Aim of this study was to descriptively assess patterns of cardiac Ga-68 FAPI uptake in consecutive patients having undergone this imaging technique for staging after cancer treatment. Furthermore, we aimed to link this tracer uptake to clinical characteristics with respect to a cardiac disease history of the study cohort.

## Methods

### Patient Enrollment

We retrospectively analyzed patients having undergone FAPI PET imaging for cancer staging at the University Hospital Essen, Germany. According to the local standard, patients receiving anti-cancer treatment undergo cardiac diagnostic work-up before chemotherapy as well as half-annually after initiation of therapy to identify potential complications at an early stage. This standardized diagnostic work-up includes assessment of medical records, assessment of NYHA state and medical history by direct questioning as well as transthoracic echocardiography. Baseline characteristics comprised age at FAPI scan, sex, history of atrial fibrillation, coronary artery disease (CAD) including a history of MI, left ventricular ejection fraction (LVEF), cardiovascular risk factors, chronic kidney disease, and cancer entity including applied specific chemo- and immunotherapy. Anti-cancer treatment included alkylating agents, antibodies, anthracycline and platin derivatives, topoisomerase inhibitors, antimetabolites, and taxanes. Radiation therapy plans were used to calculate the heart dose in patients who received radiation during the course of cancer therapy. All relevant information were collected in a self-designed encrypted database, with all patients having signed informed consent before FAPI scan. All procedures performed were in accordance with the ethical standards of the institutional review board (IRB) of the Medical Faculty of the University Duisburg-Essen and with the principles of the 1964 Declaration of Helsinki and its later amendments.

### FAPI Imaging

PET was performed 12 ± 7 minutes after injection of 140 ± 24 MBq Ga-68 FAPI-04 as previously described.^13^ Acquisitions were performed on one of the following scanners: Biograph mCT PET/computed tomography (CT) (N = 19), Biograph Vision PET/CT (N = 10), or Biograph mMR PET/MRI (N = 3), all Siemens Healthcare GmbH, Erlangen, Germany. 3D image reconstruction (2 × 2 × 2 mm voxel size) of PET data assessed by PET/MRI was performed using ordinary Poisson ordered subset expectation maximization with 3 iterations and 21 subsets, a Gaussian filter with 4.0 mm full-width at half-maximum, and a 344 × 344 image matrix. Attenuation correction of the acquired PET data was performed using a five-compartment model attenuation map calculated from fat-only and water-only Dixon-based sequences by segmentation into background, lung, fat, and soft tissue with the addition of bone using an atlas. In the case of contrast-enhanced acquired images with MRI, only PET-based image analysis was performed because of the small sample size and because no heart-specific images were available in all cases. In the case of CT imaging, low-dose CTs for the purpose of attenuation correction and morphological correlation using automated tube voltage selection (CareKV, preset 120 kV, slice thickness: 5 mm) and CareDose 4D preset with 40 mAs were performed. PET images acquired on PET/CTs were reconstructed using an ordered subset expectation maximization (OSEM) algorithm, including time-of-flight information, with 4 iterations and 8 subsets. A Gaussian filter kernel with a full width at half maximum of 4 mm was used for post-reconstruction filtering.

### Image Interpretation

In the case of focal tracer uptake in the left ventricular myocardium, a volume of interest (VOI) was defined comprising the entire focal tracer accumulation. Uptake of remote myocardium and blood pool was assessed by placing a spherical 1 cm^3^ VOI in the lateral wall of the left ventricle or the right atrium. From these VOIs, the maximum standardized uptake value (SUV_max_) and the mean SUV (average uptake in a spheric VOI of 1 cm^3^ surrounding the hottest voxel) were measured. In the case of focal tracer accumulation in the lateral wall, cardiac remote uptake has been measured by placing this spherical VOI in the septum (there was no case with tracer uptake both in the lateral wall or the right atrium). All data were analyzed using dedicated medical imaging software (syngo.via, Siemens Healthcare GmbH, Erlangen).

### Statistical Methods

Continuous variables are expressed as mean ± standard deviation unless otherwise annotated. A Kolmogorov–Smirnov test was conducted to test for normality where indicated. Non-parametric and parametric variables were compared using the Mann–Whitney *U* test and an unpaired *t* test, respectively, Chi-square test was applied to compare dichotomized variables. SUV_mean_ and SUV_max_ were used for further assessment of variables associated with remote FAPI uptake in a univariate linear regression model. To compare matched continuous variables, the 2-tailed paired Student *t* test was applied. A *P* value of less than .05 was considered statistically significant. Statistical analyses were performed using SPSS for Windows (Version 22.0, SPSS Inc., Chicago, IL, USA).

## Results

### Baseline Characteristics

From 09/2018 to 11/2019 N = 32 patients underwent PET imaging for staging after systemic anti-cancer treatment for different kinds of cancers. The majority of patients was female (17/32, 53.1%), with an overall mean age of 58.7 ± 15.0 years at time of PET scan. Left ventricular EF was preserved in the majority of patients, with an overall mean LVEF of 57.5 ± 7.3 %. The most frequent tumor entities were pancreatic cancer (18/32 (56.3%)), melanoma (2/32 (6.3%)), and osteosarcoma (2/32 (6.3%)).

### Visual and Quantitative Assessment of Myocardial FAPI Uptake in the Overall Cohort

Six out of 32 patients (18.8%) demonstrated focal myocardial FAPI tracer accumulation clearly above background. Quantitative assessment of this focal myocardial uptake in those six patients was significantly higher compared to remote myocardium (SUV_max_ 7.1 ± 4.8 vs 2.2 ± .6, *P* < .05, SUV_mean_ 5.2 ± 4.0 vs 1.6 ± .4, *P* < .05) and significantly higher than the corresponding remote myocardial uptake in patients without focal myocardial uptake (SUV_max_ 7.1 ± 4.8 vs 1.5 ± .4, *P* < .05, SUV_mean_ 5.2 ± 4.0 vs 1.2 ± .3, *P* < .05).

Also, FAPI uptake in remote myocardium was higher in patients with vs without focal myocardial tracer uptake (SUV_max_ 2.2 ± .6 vs 1.5 ± .4, *P* < .05, SUV_mean_ 1.6 ± .4 vs 1.2 ± .3, *P* < .05). Blood pool was not different between patients with vs without focal myocardial tracer uptake (SUV_max_ 2.2 ± .7 vs 2.1 ± .7, *P* = .6; SUV_mean_ 1.4 ± .4 vs 1.4 ± .5, *P* = .8).

### Association of Myocardial FAPI Uptake with Patient Characteristics and Clinical Parameters

Table 1 lists the baseline characteristics of the study cohort stratified for a visual presence of focal FAPI uptake. With respect to cancer entity and applied chemo- and immunotherapy, no significant differences regarding focal cardiac FAPI uptake were observed except for a slightly higher percentage of patients with pancreatic cancer and a history of platin/antimetabolite chemotherapy in the non-uptake group. Four out of 32 patients (12.5%) had undergone radiation therapy in the course of cancer treatment. One patient received thoracic radiation in 04/2015 and 07/2018 for local therapy of right-sided non-small cell lung cancer (right lower lobe) with a cumulative dose of only 1.04 Gy. This patient showed no focal FAPI tracer uptake. In the remaining three patients the heart dose was 0 Gy.

**Table 1:** Patient characteristics including history of cancer/anti-cancer treatment

	Overall N = 32	Visual uptake N = 6	No visual uptake N = 26	*P* value
Male sex, N	15 (46.9%)	5 (83.3%)	10 (38.5%)	.08
Age at FAPI scan, years	58.7 ± 14.9	70.8 ± 10.1	56.0 ± 14.6	.03
History of CAD, N	3 (9.4%)	3 (50%)	0	< .01
History of MI, N	2 (3.1%)	2 (33.3%)	0	.03
LVEF, %	57.5 ± 7.3	46.0±8.5	60.1 ± 4.2	< .01
Nicotine abuse, N	7 (21.9%)	1 (16.7%)	6 (23.1%)	1.0
Arterial hypertension, N	17 (53.1%)	5 (83.3%)	12 (46.2%)	.18
Diabetes, N	6 (18.8%)	2 (33.3%)	4 (15.4%)	.31
Atrial fibrillation, N	2 (3.1%)	0	2 (7.7%)	1.0
CKD, N	4 (12.5%)	2 (33.3%)	2 (7.7%)	.15
Cancer entity, N				
Pancreatic	18 (56.3%)	2 (33.3%)	16 (61.5%)	.03
Melanoma	2 (6.3%)	1 (16.7%)	1 (3.8%)	.47
Osteosarcoma	2 (6.3%)	0	2 (7.7%)	.37
Cervix	1 (3.1%)	0	1 (3.8%)	.53
Colorectal	1 (3.1%)	0	1 (3.8%)	.53
Hypopharynx	1 (3.1%)	0	1 (3.8%)	.53
Larynx	1 (3.1%)	1 (16.7%)	0	.10
Lung	1 (3.1%)	0	1 (3.8%)	.53
Nerve sheath tumor	1 (3.1%)	0	1 (3.8%)	.53
Ovarian	1 (3.1%)	0	1 (3.8%)	.53
Thyroid gland	1 (3.1%)	1 (16.7%)	0	.10
Tongue	1 (3.1%)	0	1 (3.8%)	.10
Urinary bladder	1 (3.1%)	1 (16.7%)	0	.53
Previous chemotherapy				
Antibodies	5 (15.6%)	1 (16.7%)	4 (15.4%)	.68
Anthracyclines	3 (9.4%)	0	3 (11.5%)	.26
Antimetabolites	18 (56.3%)	2 (33.3%)	16 (61.5%)	.03
Platine derivates	20 (62.5%)	2 (33.3%)	18 (69.2%)	.01
Topoisomerase inhibitors	12 (37.5%)	1 (16.7%)	11 (42.3%)	.07
Alkylating agents	3 (9.4%)	0	3 (11.5%)	.26
Taxane agents	10 (31.3%)	2 (33.3%)	8 (30.8%)	.52

*CAD*, coronary artery disease; *dx*, diagnosis; *MI*, myocardial infarction; *LVEF*, left ventricular ejection fraction; *CKD*, chronic kidney disease; *BMI*, body mass index

**Figure 1:**
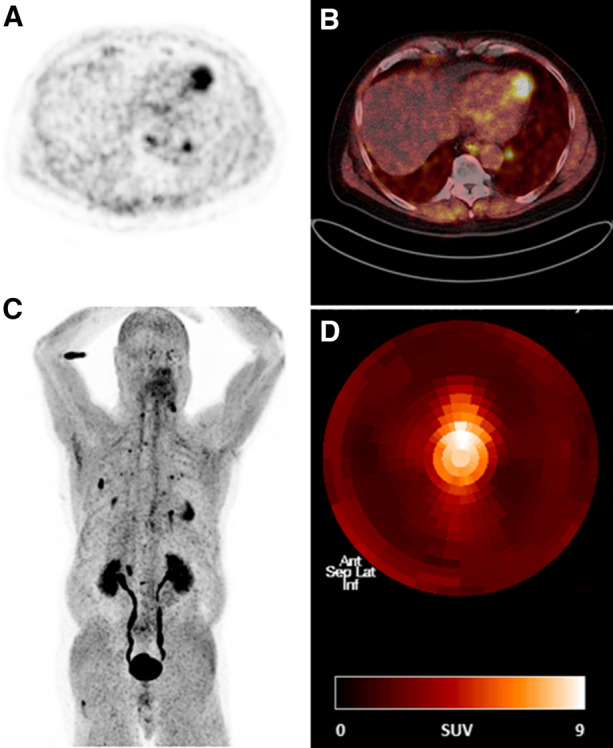
Localized apical FAPI uptake in a patient (patient #29) with papillary thyroid cancer. The whole-body FAPI scan reveals FAPI uptake in cervical lymph nodes as well as suspicion of pulmonary metastases. (A) Trans-axial slice of Ga-68 FAPI PET. (B) Fusion of trans-axial Ga-68 FAPI PET and low-dose CT. (C) Maximum intensity projection of the whole body PET. (D) Polar map demonstrating spatial Ga-68 FAPI uptake in the left ventricular myocardium

**Figure 2:**
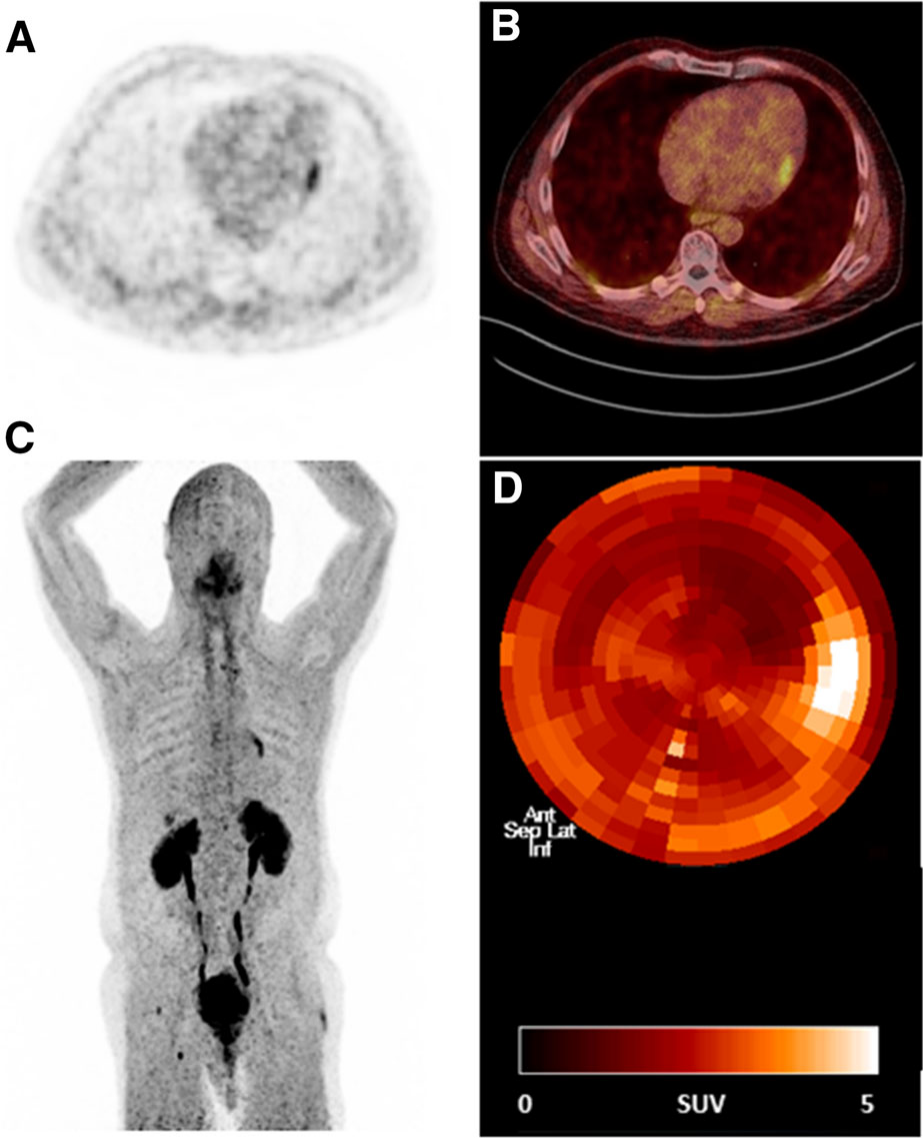
Localized lateral FAPI uptake in a patient (patient #22) without a known history of coronary artery disease. FAPI scan was performed for staging after treatment of urinary bladder carcinoma without evidence of recurrency but reactive FAPI uptake due to enthesopathic changes (both hips, right ischial tuberosity). (**A**) Trans-axial slice of Ga-68 FAPI PET, (**B**) fusion of trans-axial Ga-68 FAPI PET and low-dose CT, (**C**) maximum intensity projection of the whole-body PET, (**D**) polar map demonstrating spatial Ga-68 FAPI uptake in the left ventricular myocardium

Our data suggest that patients with focal FAPI uptake were older (70.8 ± 10.1 vs 56.0 ± 14.6 years, *P* = .03), with a lower LVEF (46.0 ± 8.5 vs 60.1 ± 4.2; *P* < .01) and a higher percentage of significant CAD (50.0% vs 0%; *P* < .01) compared to non-uptake patients. The exact location of visually detectable tracer uptake in the 6 patients is reported in Table 2. Looking at the patients with a known diagnosis of CAD it appears that the location of the primarily treated or affected myocardium (coronary region treated by percutaneous intervention or location of MI) in these 3 patients correlated well with the area of visual FAPI accumulation. In opposite, no patient in the FAPI-negative group (patients without localized uptake) showed a history of CAD or MI (*P* < .01 and *P* = .03 for CAD and MI, respectively). Figures 1 and 2 depict representative examples of 2 patients with localized myocardial FAPI uptake.

**Table 2:** Specific patterns of FAPI uptake, depending on a history of CAD/MI with the primary treated coronary vessel(s) with PTCA/DES

Patient ID	Tracer uptake	CAD	MI	Location of MI; treated coronary vessel	Time between infarction/intervention and imaging
2	Posterior wall (main uptake) Antero-septal	Yes	No	PTCA/DES RCA 2012	
7	Antero-septal	Yes	Yes	PTCA/DES proximal LAD for MI 09/2019	MI LAD: 5 days
10	LV circumferential	Yes	Yes	PTCA/DES for MI LAD/RCX 2012 and elective PTCA/DES RCA 2018	MI LAD/RCX: 2713 days
22	Lateral	No	No	–	
29	Apical	No	No	–	
31	LV	No	No	–	

*CAD* coronary artery disease; *MI*, myocardial infarction; *LV*, left ventricle; *PTCA*, percutaneous transluminal coronary angiography; *DES*, drug-eluting stenting; *LAD*, left anterior descending artery; *RCX*, ramus circumflexus; *RCA*, right coronary artery

Univariate regression showed a weak, but significant correlation of the clinical variables CAD, MI, and age with remote SUV_mean_ (*R*^2^ = .16, *P* = .03, *R*^2^ = .14, *P* = .04, and *R*^2^ = .15, *P* = .04, respectively), whereas LVEF was strongly correlated with remote uptake (*R*^2^ = .74, *P* < .01) (Table 3).

**Table 3:** Univariate analysis assessing impact of clinical covariates on remote FAPI uptake (SUV_mean_)

	*R*^2^	*P* value
CAD	.16	**.03**
Age	.15	**.04**
LVEF	.74	**< .01**
Sex	.01	.65
MI	.14	**.04**
Nicotine	.19	.17
Hypertension	.04	.29
Diabetes	.01	.68
CKD	.01	.97
AF	.10	.08

Bold *P* values indicate statistical significance

*R*, correlation coefficient; *SUV*, standardized uptake value; other abbreviations as in Table 1

## Discussion

This is the first in-human study describing the pattern of cardiac FAPI uptake in a cohort of patients who underwent Ga-68 FAPI PET imaging for cancer staging. The main findings are that patients with visual detectable (focal) tracer uptake (6/32 patients, 18.8%) were older, with a higher prevalence of CAD/MI and lower LVEF. In patients with CAD, the visual tracer uptake seemed to correlate with the area of primarily diseased myocardium. In patients with increased uptake in certain myocardial areas, an increased uptake was also found in the remote myocardium, while no difference in blood pool activity was detected. Remote myocardial uptake was correlated to CAD, age, and LVEF, the latter showing a highly significant correlation.

### First In-Human Experience Assessing Cardiac Localized FAPI Uptake and Its Correlation with Heart Disease and Clinical Comorbidities

Fibroblast activation protein is not expressed in normal cardiac fibroblasts^16^; nevertheless, cardiac FAP activation has been reported under various pathologic conditions in animal models.^5^,^17^ The possibility of detecting fibroblast activity by FAPI PET/CT after MI has already been shown in a murine model.^10^ In our present in-human experience on cardiac FAPI tracer accumulation we could demonstrate that in an unselected cohort of individuals with malignancy about 20% of subjects showed distinct cardiac tracer accumulation. This raises the question if this localized tracer accumulation could be attributed to prior myocardial injury due to the chemotherapy applied in the course of cancer treatment as preliminary data suggested a potential role of chemo-therapy-induced cardiotoxicity on myocardial FAPI enrichment.^18^ Despite a medical history of several chemotherapeutic drugs applied in the study cohort we were not able to link the chemotherapy regimens to distinct FAPI uptake. Detailed analysis of the 20% of patients of the present study with localized tracer accumulation revealed that those patients were older, with a lower LVEF and higher prevalence of CAD. This goes in line with clinical and preclinical data suggesting that LV function,^19^,^20^ age^21^, and especially CAD/MI^10^,^17^ are associated with myocardial fibrosis. With respect to localized tracer uptake we hypothesize that lower EF and higher age show a significant interaction with CAD, as patients with CAD were older and had a significantly lower EF (73.0 vs 57.2 years and 46.0 vs 60.1%), respectively. In the three patients with CAD, the area of mainly diseased myocardium correlated well with the localized FAPI uptake in those patients. This is of particular relevance considering that following myocardial injury due to myocardial ischemia, FAP-α has been shown to be enriched in adjacent myocardium (close to infarcted myocardium, with reduced function and perfusion) compared to remote myocardium (preserved function and perfusion) in a pig model of myocardial infarction.^5^ This leads to the hypothesis that visualization of FAP expression could help to assess the extent of myocardial damage following MI in progressive CAD and to further assess the extent of cardiac re-modeling following such ischemic events. Large controlled studies are warranted to investigate this hypothesis.

### Potential Significance of FAPI Uptake in Remote Areas

We demonstrate that fibroblast activation in the remote myocardium is also significantly increased in patients with focally increased uptake of certain myocardial areas. This points at left ventricular remodeling processes in those patients besides the localized uptake areas. At the moment we cannot draw a final conclusion on the mechanisms behind higher fibroblast activation in remote areas as the patient number in the present study was low. We speculate that potentially a generalized myocardial injury, as it is the case in microvascular dysfunction present in CAD, could be causative for this phenomenon. This cannot be finally proven at the moment as there are not conclusive histopathological studies validating cardiac FAPI uptake with fibroblast activation as it is the case for FAPI uptake in malignancy.^9^,^10^ Fibroblast activation may also play a role in generalized fibrotic remodeling, as previously shown in heart failure, where the deposition of extracellular matrix proteins increasing myocardial stiffness^1^ is primarily driven by cardiac fibroblasts.^22^ As fibrosis is involved in the disruption of myocardial excitation-contraction coupling this is suggested to play a key role in the development of both systolic and diastolic heart failure.^22^ Especially looking at the complex entity of heart failure with preserved EF, this imaging modality might bear the potential of increasing diagnostic yield, further guiding specific therapy in the future.

### Limitations

This is a retrospective study of FAPI PET in a heterogeneous population of patients. Therefore, the data must be treated with caution and do not allow to draw general conclusions. Some limitations have to be mentioned. First, despite preclinical work showing highly specific binding to FAP,^10^ we cannot conclusively prove that myocardial FAPI accumulation is specific for FAP expression or fibroblast activation as we are not able to provide histopathological validation or blocking study data. We acknowledge the limitation of not having profound data on diastolic function in our cohort in order to further investigate the impact of this confounder on our results. As no routine invasive or non-invasive screening for CAD has been performed we are not able to entirely rule out unidentified myocardial ischemia as potential confounder for localized FAPI uptake in patients without a known history of CAD. Last, as this is a pilot study on cardiac FAPI uptake, scans were assessed on a consensus decision of the nuclear medicine specialists. As the analysis was clearly defined and highly standardized requiring only minimal user interaction, a high interobserver reliability can be assumed.

## Conclusion

This is the first report to assess cardiac FAPI uptake in cancer patients. Higher age, a history of CAD/MI as well as impaired LVEF seem to be associated with increased localized uptake. These results point towards the potential role of FAPI imaging to assess myocardial localized as well as generalized injury. Further research efforts are needed to clearly link myocardial FAPI uptake to myocardial fibroblast activation. Finally, studies are warranted to assess if FAPI uptake might be used to risk-stratify patients with respect to a progression of CAD or left ventricular remodeling.

### New Knowledge Gained

This pilot study demonstrates that cardiac fibroblast activity can be assessed by Ga-68 FAPI PET imaging. Fibroblast activity correlated with CAD, MI, age, and especially LVEF, pointing at the potential of this technique to significantly improve the understanding of structural/fibrotic remodeling processes in cardiac pathophysiology.

## Electronic supplementary material

Below is the link to the electronic supplementary material.

Electronic supplementary material 1 (DOCX 159 kb)

Electronic supplementary material 2 (M4A 4056 kb)

Electronic supplementary material 3 (PPTX 560 kb)

## Abbreviations

CAD: Coronary artery disease
FAP(I): Fibroblast activation protein (inhibitor)
Ga-68: Gallium-68
LVEF: Left ventricular ejection fraction
MI: Myocardial infarction
MRI: Magnetic resonance imaging
PET: Positron emission tomography
CT: Computed tomography
SUV: Standardized uptake volume
VOI: Volume of interest
