# Supplementary material for: Cardiac fibroblast activation detected by Ga-68 FAPI PET imaging as a potential novel biomarker of cardiac injury/remodeling
Source: J Nucl Cardiol. 2020 Sep 25;28(3):812–21. doi: 10.1007/s12350-020-02307-w (PMC8249249; doi:10.1007/s12350-020-02307-w)
Supplement: Supplementary file 1 — Electronic supplementary material 1 (DOCX 159 kb) [file 12350_2020_2307_MOESM1_ESM.docx]

**Cardiac fibroblast activation detected by Ga-68 FAPI PET imaging as a potential novel biomarker of cardiac injury/remodeling**

Siebermair J^1,3^, Köhler MI^1^, Kupusovic J^1^, Nekolla SG^2,3^, Kessler L^4^, Ferdinandus J^4^, Guberina N^5^, Stuschke M^5^, Grafe H^4^, Siveke JT^6,7^, Kochhäuser S^1^, Fendler WP^4^, Totzeck M^1^, Wakili R^1,3^, Umutlu L^8^, Herrmann K^4^, Rassaf T^1^, Rischpler C^4^

^1^ Department of Cardiology and Vascular Medicine, West German Heart and Vascular Center Essen, University of Essen Medical School, University Duisburg-Essen, Hufelandstr. 55, 45147 Essen, Germany

^2^ School of Medicine, Department of Nuclear Medicine, Technische Universität München, Germany

^3^ DZHK (Deutsches Zentrum für Herz-Kreislauf-Forschung e.V.), Partner Site Munich Heart Alliance, Munich, Germany

^4^ Department of Nuclear Medicine, University Hospital Essen, Medical Faculty, University of Duisburg-Essen, Hufelandstr. 55, 45147 Essen, Germany

^5^ Department of Radiotherapy, University Hospital Essen, University Duisburg-Essen, Essen, Germany.

^6^ Institute for Developmental Cancer Therapeutics, West German Cancer Center, University Hospital Essen, Hufelandstrasse 55, 45147, Essen, Germany

^7^ Division of Solid Tumor Translational Oncology, German Cancer Consortium (DKTK, partner site Essen) and German Cancer Research Center, DKFZ, Im Neuenheimer Feld 280, 69120, Heidelberg, Germany

^8^ Institute for Diagnostic and Interventional Radiology and Neuroradiology, University Hospital Essen, University of Duisburg-Essen, Hufelandstr. 55, 45147 Essen, Germany

**Brief title:** Cardiac fibroblast activation detected by Ga-68 FAPI PET

**Word count: 4258** (including tables, figure legends, references)

**Online supplement:**

Fig. S1.

Circumferential, myocardial FAPI uptake in the LV (a, b, d) in a patient (patient #10) with pancreatic cancer, with intense uptake especially in the pancreatic tail (c). Additionally, PET imaging shows intense liver uptake as well as suspicion of peritoneal carcinomatosis.


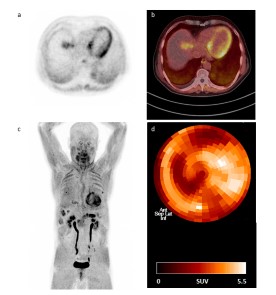


Fig. S2.

Myocardial FAPI uptake predominantly in the septum/posteroseptal (a, b, d) in a patient (patient #2) with pancreatic cancer. Besides pancreatic uptake mainly in the tail there is suspicion of multiple metastases in the left scapula, the skull, left ribs and in the peri-clavicular lymph nodes (c).


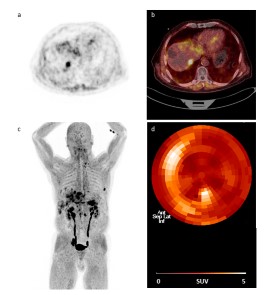


Fig. S3.

Antero-septal FAPI uptake of the LV (a, b, d) in a female patient (patient #7) with melanoma. In addition, there is suspicion of multiple, FAPI positive metastases of the brain and lungs (c).


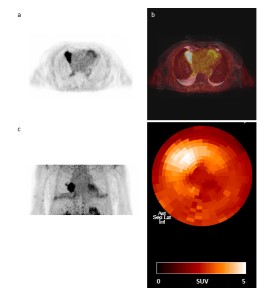


Fig. S4.

Significant antero-lateral FAPI uptake in the left ventricle (a, b, d) in a male patient (patient #31) with larynx carcinoma and multiple cervical lymph node metastases (c).


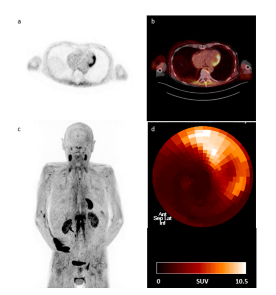


Fig. S5.

Significant pancreatic and hepatic tracer uptake in a patient (patient #8) with pancreatic cancer (c), without evidence of cardiac FAPI uptake (a, b).
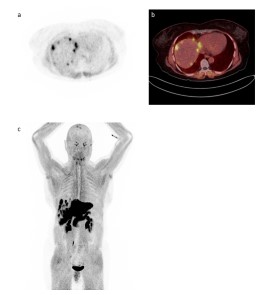


Fig S6.

Intense FAPI uptake in the pancreatic tail and retroperitoneal lymph nodes in a patient (patient #25) with pancreatic cancer (c), without cardiac tracer uptake (a, b).


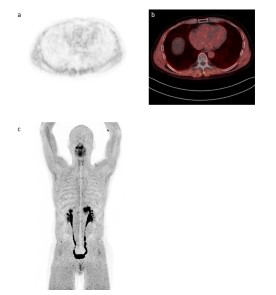


A: Trans-axial slice of Ga-68 FAPI PET

B: Fusion of trans-axial Ga-68 FAPI PET and low-dose CT

C: Maximum intensity projection of the whole body PET

D: Polar map demonstrating spatial Ga-68 FAPI uptake in the left ventricular myocardium
