# Supplementary material for: Cardiac fibroblast activation detected by Ga-68 FAPI PET imaging as a potential novel biomarker of cardiac injury/remodeling
Source: J Nucl Cardiol. 2020 Sep 25;28(3):812–21. doi: 10.1007/s12350-020-02307-w (PMC8249249; doi:10.1007/s12350-020-02307-w)
Supplement: Supplementary file 3 — Electronic supplementary material 3 (PPTX 560 kb) [file 12350_2020_2307_MOESM3_ESM.pptx]

## Slide 1
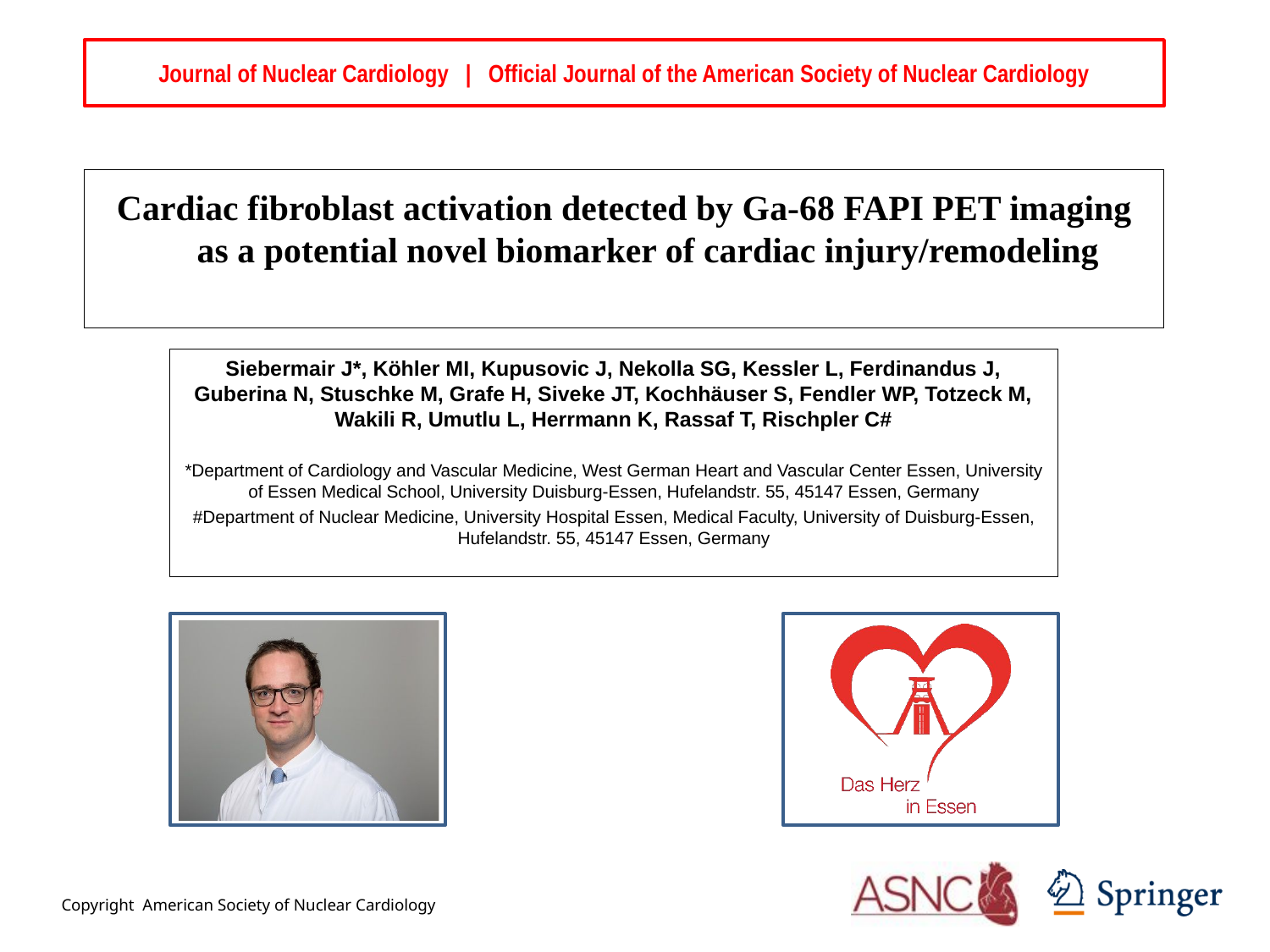

Journal of Nuclear Cardiology | Official Journal of the American Society of Nuclear Cardiology
# Cardiac fibroblast activation detected by Ga-68 FAPI PET imaging as a potential novel biomarker of cardiac injury/remodeling
Siebermair J*, Köhler MI, Kupusovic J, Nekolla SG, Kessler L, Ferdinandus J, Guberina N, Stuschke M, Grafe H, Siveke JT, Kochhäuser S, Fendler WP, Totzeck M, Wakili R, Umutlu L, Herrmann K, Rassaf T, Rischpler C#
*Department of Cardiology and Vascular Medicine, West German Heart and Vascular Center Essen, University of Essen Medical School, University Duisburg-Essen, Hufelandstr. 55, 45147 Essen, Germany
#Department of Nuclear Medicine, University Hospital Essen, Medical Faculty, University of Duisburg-Essen, Hufelandstr. 55, 45147 Essen, Germany
Head shot of author
required
Copyright American Society of Nuclear Cardiology

## Slide 2
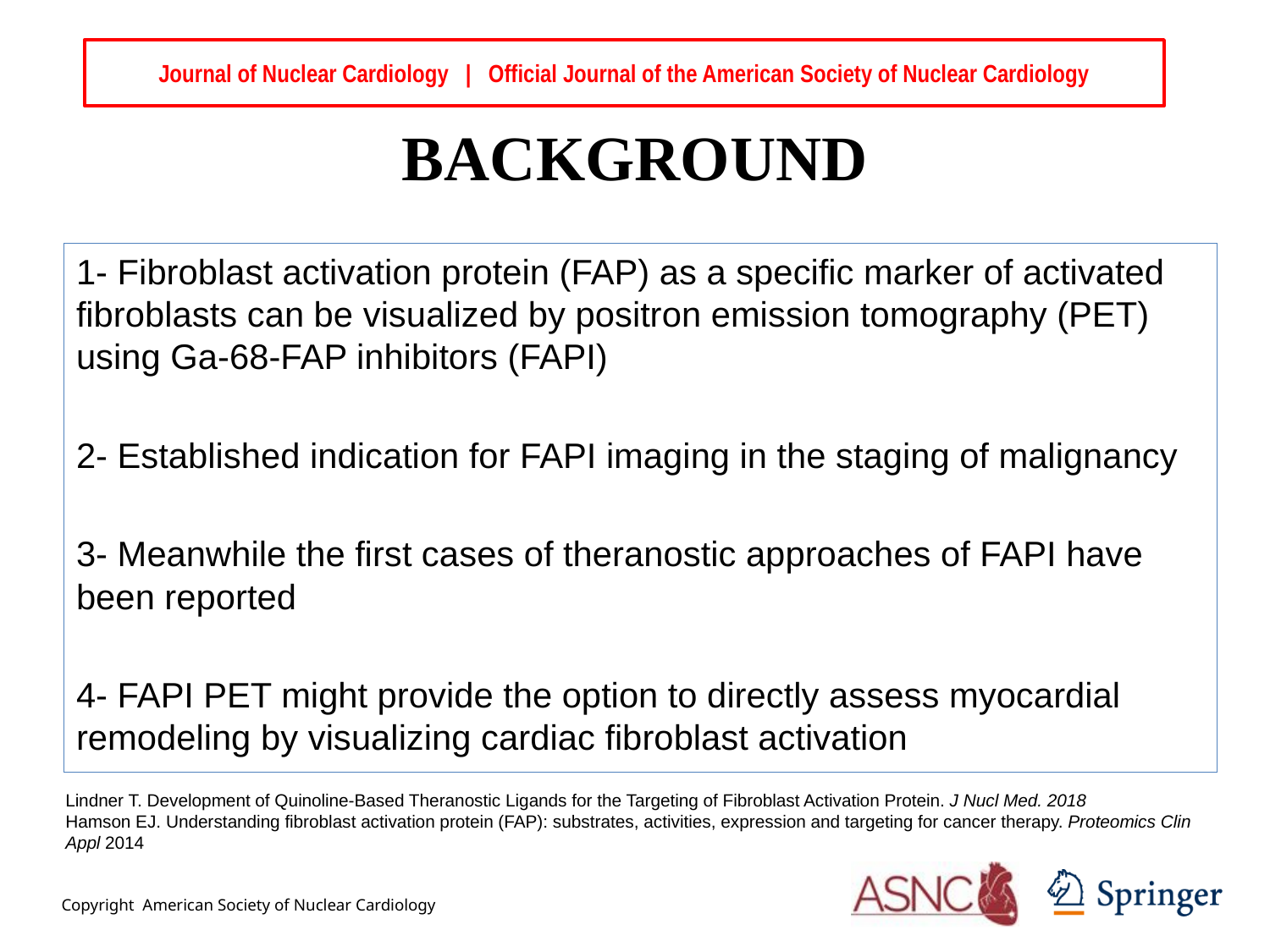

Journal of Nuclear Cardiology | Official Journal of the American Society of Nuclear Cardiology
# BACKGROUND
1- Fibroblast activation protein (FAP) as a specific marker of activated fibroblasts can be visualized by positron emission tomography (PET) using Ga-68-FAP inhibitors (FAPI)
2- Established indication for FAPI imaging in the staging of malignancy
3- Meanwhile the first cases of theranostic approaches of FAPI have been reported
4- FAPI PET might provide the option to directly assess myocardial remodeling by visualizing cardiac fibroblast activation
Lindner T. Development of Quinoline-Based Theranostic Ligands for the Targeting of Fibroblast Activation Protein. J Nucl Med. 2018
Hamson EJ. Understanding fibroblast activation protein (FAP): substrates, activities, expression and targeting for cancer therapy. Proteomics Clin Appl 2014
Copyright American Society of Nuclear Cardiology

## Slide 3
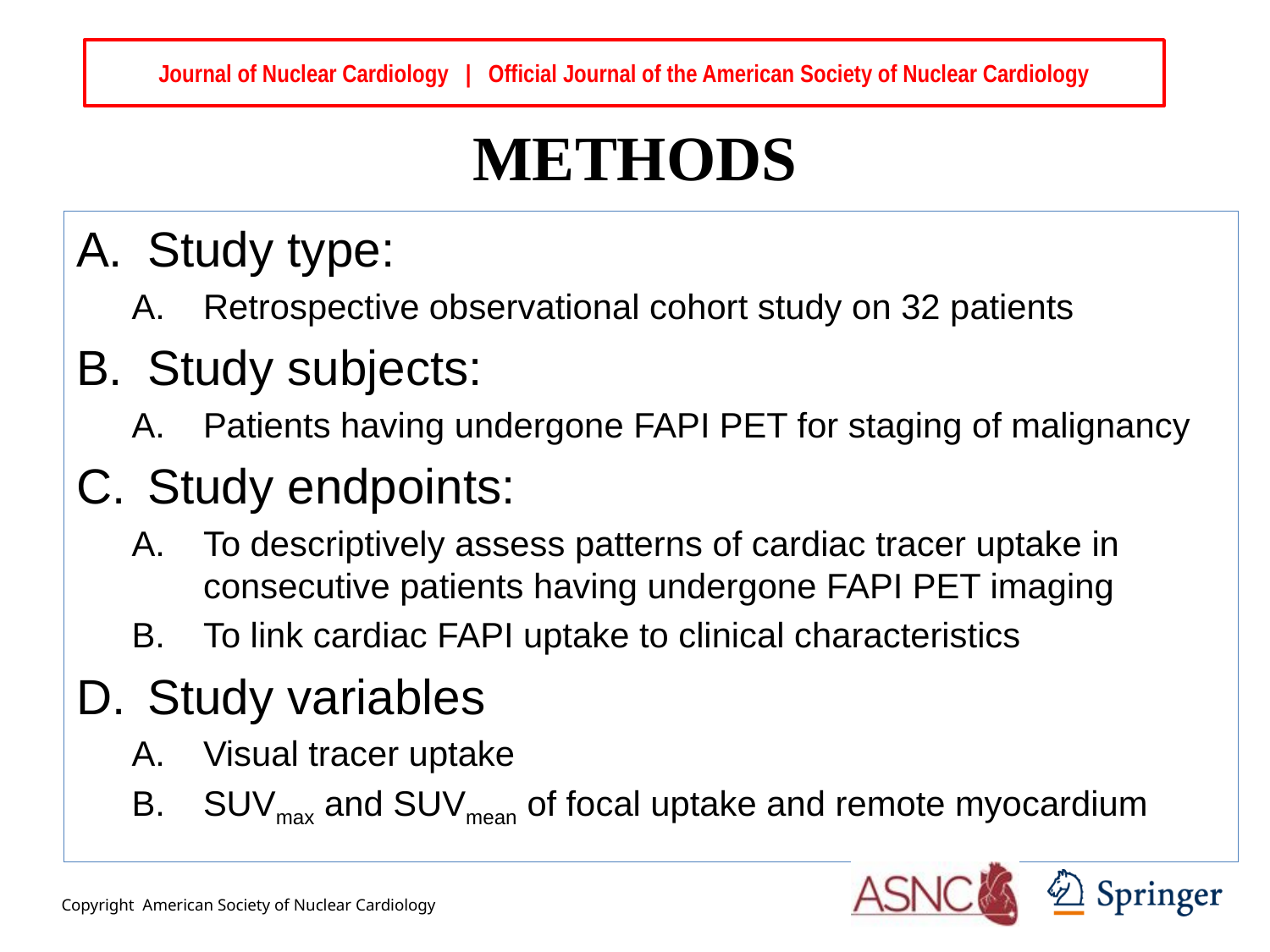

Journal of Nuclear Cardiology | Official Journal of the American Society of Nuclear Cardiology
# METHODS
Study type:
Retrospective observational cohort study on 32 patients
Study subjects:
Patients having undergone FAPI PET for staging of malignancy
Study endpoints:
To descriptively assess patterns of cardiac tracer uptake in consecutive patients having undergone FAPI PET imaging
To link cardiac FAPI uptake to clinical characteristics
Study variables
Visual tracer uptake
SUVmax and SUVmean of focal uptake and remote myocardium
Copyright American Society of Nuclear Cardiology

## Slide 4
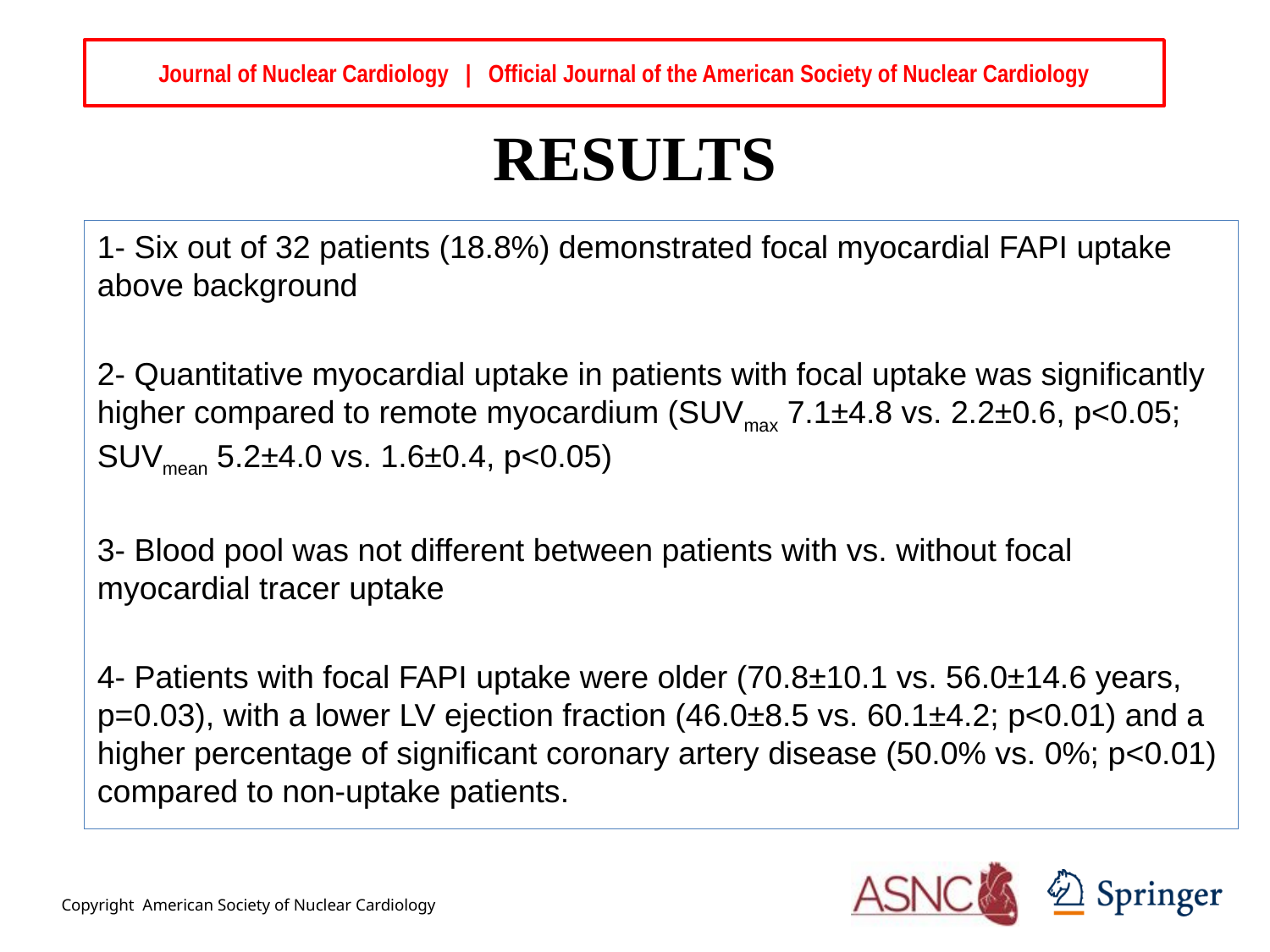

Journal of Nuclear Cardiology | Official Journal of the American Society of Nuclear Cardiology
# RESULTS
1- Six out of 32 patients (18.8%) demonstrated focal myocardial FAPI uptake above background
2- Quantitative myocardial uptake in patients with focal uptake was significantly higher compared to remote myocardium (SUVmax 7.1±4.8 vs. 2.2±0.6, p<0.05; SUVmean 5.2±4.0 vs. 1.6±0.4, p<0.05)
3- Blood pool was not different between patients with vs. without focal myocardial tracer uptake
4- Patients with focal FAPI uptake were older (70.8±10.1 vs. 56.0±14.6 years, p=0.03), with a lower LV ejection fraction (46.0±8.5 vs. 60.1±4.2; p<0.01) and a higher percentage of significant coronary artery disease (50.0% vs. 0%; p<0.01) compared to non-uptake patients.
Copyright American Society of Nuclear Cardiology

## Slide 5
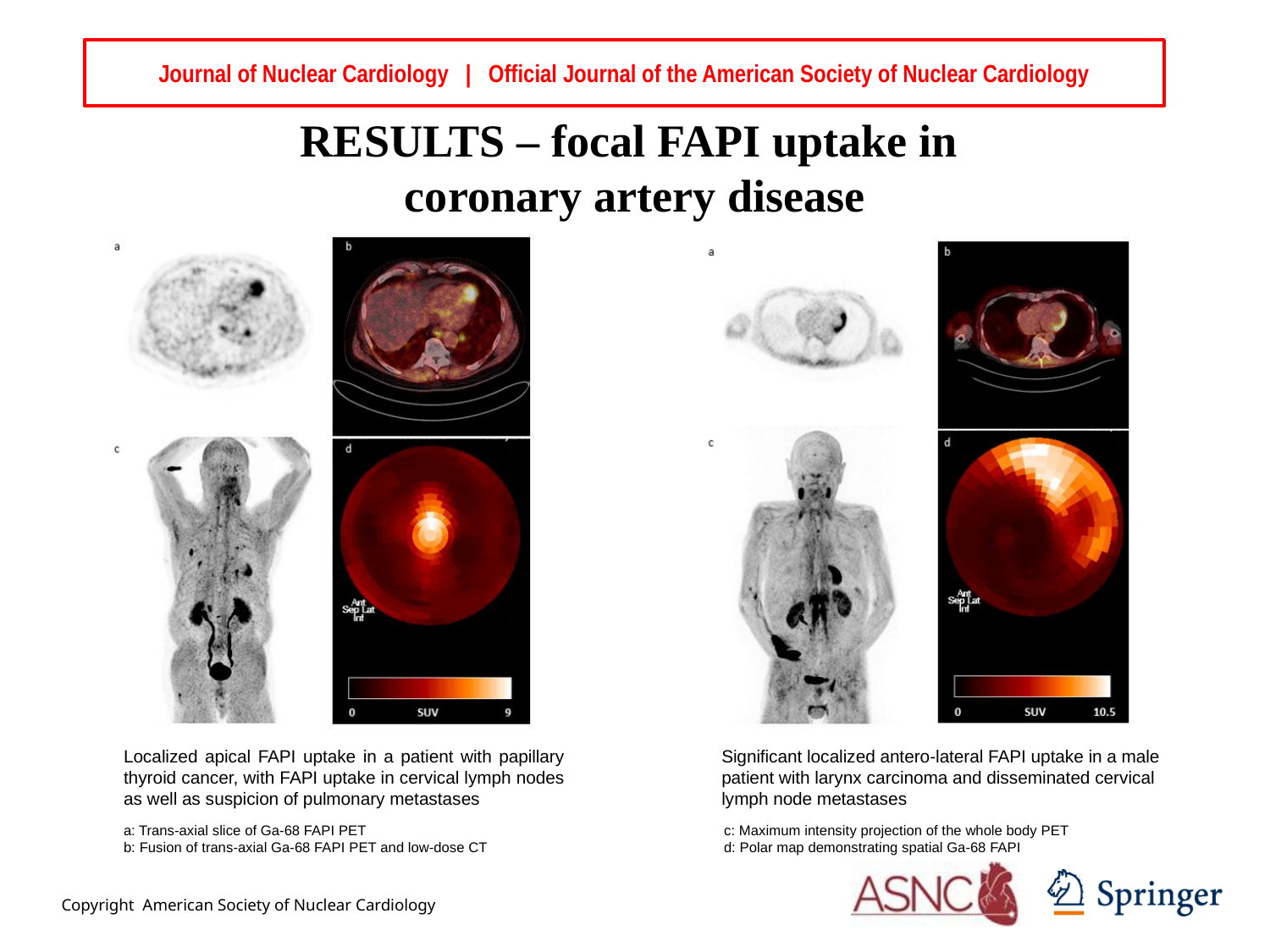

Journal of Nuclear Cardiology | Official Journal of the American Society of Nuclear Cardiology
# RESULTS – focal FAPI uptake in coronary artery disease
Localized apical FAPI uptake in a patient with papillary thyroid cancer, with FAPI uptake in cervical lymph nodes as well as suspicion of pulmonary metastases
Significant localized antero-lateral FAPI uptake in a male patient with larynx carcinoma and disseminated cervical lymph node metastases
a: Trans-axial slice of Ga-68 FAPI PET
b: Fusion of trans-axial Ga-68 FAPI PET and low-dose CT
c: Maximum intensity projection of the whole body PET
d: Polar map demonstrating spatial Ga-68 FAPI
Copyright American Society of Nuclear Cardiology

## Slide 6
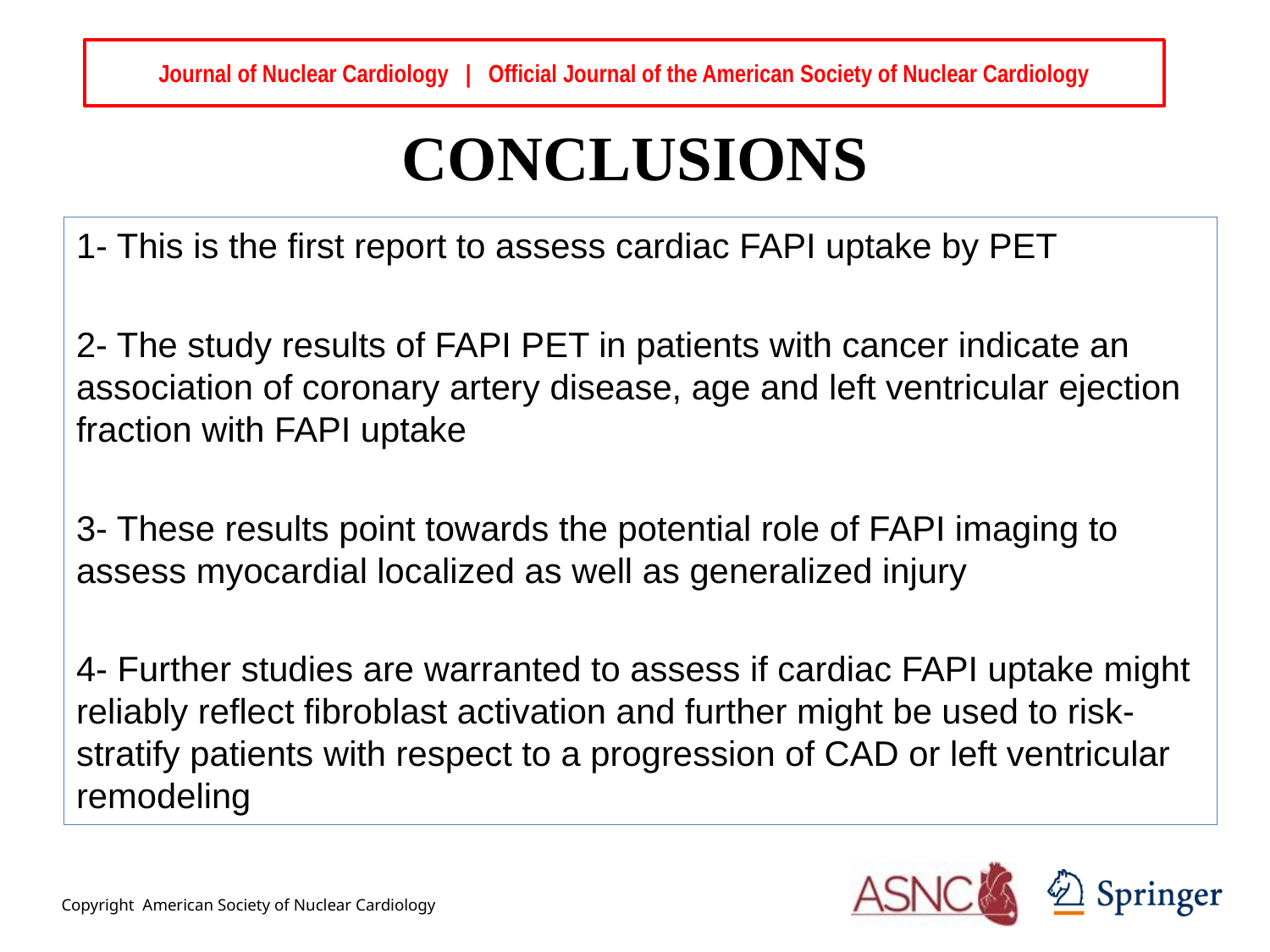

Journal of Nuclear Cardiology | Official Journal of the American Society of Nuclear Cardiology
# CONCLUSIONS
1- This is the first report to assess cardiac FAPI uptake by PET
2- The study results of FAPI PET in patients with cancer indicate an association of coronary artery disease, age and left ventricular ejection fraction with FAPI uptake
3- These results point towards the potential role of FAPI imaging to assess myocardial localized as well as generalized injury
4- Further studies are warranted to assess if cardiac FAPI uptake might reliably reflect fibroblast activation and further might be used to risk-stratify patients with respect to a progression of CAD or left ventricular remodeling
Copyright American Society of Nuclear Cardiology
